# The Influence of Nationality and Socio-demographic Factors on Urban Slum Dwellers’ Threat Appraisal, Awareness, and Protective Practices against COVID-19 in Thailand

**DOI:** 10.4269/ajtmh.21-1096

**Published:** 2022-05-23

**Authors:** Thi Phuoc Lai Nguyen, Siwarat Pattanasri

**Affiliations:** ^1^ Department of Development and Sustainability, School of Environment, Resources and Development, Asian Institute of Technology, Klong Luang, Thailand;; ^2^ Department of Public Works and Town & Country Planning, National and Regional Planning Bureau, Bangkok, Thailand

## Abstract

This study aimed to analyze the influence of ethnicity and other demographic and social factors on urban slum dwellers’ threat appraisal, awareness, and protective practices against COVID-19. It was conducted via 20 semi-structured interviews and 453 questionnaires for different ethnic groups from Thailand, Myanmar, Laos, and Cambodia in the slum communities of Khlong Toei, Bangkok—the largest slum in Thailand. A phenomenological approach was used to analyze the semi-structured interviews to understand dwellers’ lived experiences and behaviors regarding COVID-19. The questionnaire data were analyzed using descriptive statistics and a multiple regression model. The main findings in this study were that age (elderly people), gender (female), nationality (foreign migrant groups), and type of residential occupancy (living in unoccupied spaces, under tollways, and by railroads) were significant risk factors for vulnerability to COVID-19. Type of residential occupancy and occupation (daily wage workers) were risk factors for severity of COVID-19. Higher education and female gender were factors influencing COVID-19 awareness in all ethnic groups; women tended to practice COVID-19 protection guidelines better than men. Foreign ethnic groups and daily wage workers also performed better in COVID-19 protection practices than other groups. This study appeals for urgent intervention and special assistance from development organizations, the government, and society to ensure slum communities’ access to clean water, sanitation, and health care, using dwellers’ sociodemographic characteristics and ethnicity to help enhance their threat appraisal capacity and coping strategies with regard to the pandemic.

## INTRODUCTION

Approximately 20% of the population in the Bangkok metropolis of Thailand live in slums. Slums are defined as “a wide range of low-income settlements and/or/with poor human living conditions.”^1, p.8^ Rapid economic growth in developing countries has brought about social disparities and the growth of the slum population.^2^ Slums in Bangkok city are the living places of many poor migrants from the Thailand poor countryside and other southeast Asian neighboring countries. These migrants are low-paid workers and have no access to public housing. Many slum communities in Bangkok still use unsafe water from rivers with a quality that is less than standard for drinking and domestic use. The water, sanitation, and hygiene (WASH) conditions of slum communities do not meet standards. Women, and indigenous and disabled people in slums also face inequality in access to WASH and health services.^3^ The spread of COVID-19 has affected more than 3.8 million vulnerable Thai families, especially those living in urban slum communities. Slum dwellers are the most vulnerable to COVID-19 because of their unstable economic condition, limited access to social welfare, poor housing, overcrowded neighborhoods, and polluted environment.

The Thai government enforced a lockdown and social isolation for many months in 2020 and 2021 to limit the spread of COVID-19. These measures have been devastating for dwellers in urban slums, where physical space is scarce, and the majority of these people rely on daily wage work for survival. Social distancing is impossible in urban slums.^4^

This study aimed to examine 1) how urban slum dwellers in a metropolis such as Bangkok, Thailand, perceive their vulnerability to and the severity of COVID-19; 2) their awareness of and practices of protection against COVID-19 transmission; and how nationality, social, and demographic factors affect urban slum dwellers’ threat appraisal of, awareness of, and protective practice against COVID-19.

## METHODS

### Selection of the study area.

**Figure 1.**
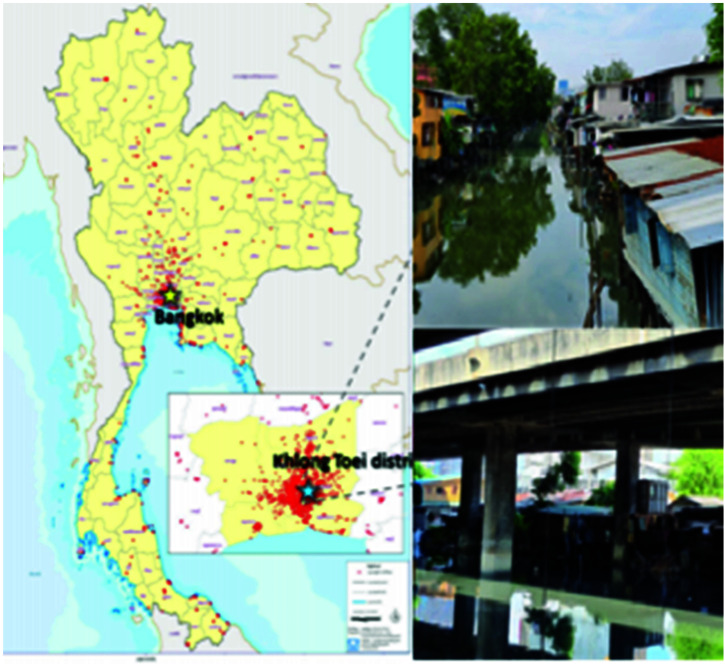
Khlong Toei slum communities in the Khlong Toei District, Bangkok, Thailand. This figure appears in color at www.ajtmh.org.

Slum communities in Khlong Toei, Bangkok, Thailand, were selected purposefully as the study area. These are the largest slums in Thailand, with 49,225 households and more than 100,000 people. Most of those who live there are seasonal workers in Khlong Toei port and the largest fresh and retail markets in Bangkok. Their WASH conditions are likely poor. They use low-quality drinking water conserved in tanks and vending machines with high levels of bacteria. The Lock 1-2-3 and Ban Guay sub-communities were selected in which to conduct interviews and complete survey questionnaires. Both communities have a high density of households and are placed along the canal and under tollway roads (Figure 1).

### Data collection.

Data collection was carried out using semi-structured interviews and questionnaires. The surveys were conducted with 453 respondents. Respondents were selected randomly from two selected sub-communities by using Slovin’s formula and the snowball sampling method. These methods are suitable for hidden populations, particularly in slum communities with little information or unknown population behaviors, and anonymous population census data.^5^ To cover different socioeconomic, ethnic, and cultural characteristics of the entire slum community population, other data were acquired during sampling, including nationality, age range, gender, and occupation. The population list of Khlong Toei slum communities was obtained from the National Statistical Office of Thailand. Semi-structured interviews were performed further with 20 selected respondents who participated in the survey. The selection of interviewees was made purposefully based on their personal information given during administration of the questionnaire. Both semi-structured interviews and questionnaires were conducted in the local languages.

### Data analysis.

Mixed qualitative and quantitative data analysis methods were applied in the study. Data from the interviews and questionnaires were treated anonymously. In the qualitative data analysis method, a phenomenological approach^6^ was used to discover slum dwellers’ lived experiences and behaviors with regard to COVID-19. Themes were generated for analyzing significant statements. During quantitative data analysis, statistical analysis was used to analyze questionnaire data. Descriptive statistics were used to describe respondents’ perceptions of vulnerability to, severity of, awareness of, and practices against contracting COVID-19. Multiple regression analysis was performed to determine factors influencing respondents’ perception of vulnerability, severity, awareness, and practices. A model was developed to explain the relationships among multiple independent variables, including age, gender, education, ethnicity, marital status, residential occupancy, and occupation, and each dependent variable (perceived vulnerability, perceived severity, awareness, and practices). The multiple regression equation

$$Y_{i}=\beta_{0}+\beta_{1}X_{1}+\beta_{2}X_{2}+\ldots +\beta_{19}X_{19}+\varepsilon$$

was used, where *Y_i_* is the dependent variable of the regression; *X*_1_, *X*_2_, . . . *X*_19_ is the independent variable of the regression, and β_0_ is the intercept term.

## RESULTS

### Sociodemographic characteristics of respondents.

Table 1 depicts the diversity of sociodemographic characteristics of the slum community in the study area. There is an even distribution of respondents among age groups, gender, and education. Many different ethnic groups living in the slum areas in Bangkok are from Thailand, Myanmar, Laos, and Cambodia. The major population is Thai, and these individuals come from other rural, remote, impoverished provinces. Most of them are small sellers, daily wage workers, or unemployed. Consequently, many people are homeless or squatters, and they live in unoccupied spaces or in houses with unclean, unsafe conditions, with water leaks and poor ventilation, which lead to mold, mites, and other allergens associated with poor health. As one man shared, “*I live under a tollway bridge. I do not have my own toilet. I must use the canal or ask for the help of my friend*.”

**Table 1:** Sociodemographic characteristics of respondents (*N *= 453)

Characteristic	No. of respondents	Frequency, %
Age, y		
15–17	80	17.66
18–35	130	28.70
36–69	136	30.02
> 70	107	23.62
Gender		
Male	220	48.57
Female	233	51.43
Education		
Illiterate	79	17.44
Primary school	145	32.01
Secondary school	112	24.72
Tertiary school	92	20.31
Other	25	5.52
Nationality		
Thailand	401	88.52
Myanmar	32	7.06
Laos	8	1.77
Cambodia	12	2.65
Marital status		
Single	195	43.05
Married	195	43.05
Separated	13	2.87
Co-living	25	5.52
Widow	25	5.52
Residential occupancy		
Own home	54	11.92
Squatter	216	47.68
Renter	106	23.40
Living with host family	21	4.64
Other	56	12.36
Occupation		
Small seller	65	14.35
Daily wage worker	153	33.77
Unemployed	101	22.30
Student	81	17.88
Employee	24	5.30
Other	29	6.40

Approximately 100 households that do not have access to tap water must rent water from their neighbors at very high prices. During the pandemic, most people in the slums have waited for donations of drinking water and food from both government and nongovernmental organizations.

### Slum dwellers’ perceived vulnerability to and severity of COVID-19.

A high percentage of respondents understood their vulnerability to COVID-19 exposure and infection (Table 2). Nearly 50% of respondents believed their poor health would prevent them from coping with COVID-19 if infected. They considered their working environment unsafe and knew they were exposed to risks. One Thai male respondent said, “*I am easily infected with COVID-19 because of my chronic disease*.” A Thai women added, “*Many elderly adults and children in my family are easily affected by COVID-19*.”

**Table 2:** Urban slum dwellers’ perceived vulnerability to and severity of COVID-19

Perceived vulnerability and severity	No. of agreed respondents	Frequency, % (*N* = 453)
Perceived vulnerability		
Poor health	205	45.25
Unsafe and exposed work environment	225	49.67
Many children and elderly people in the family	295	65.12
House condition not fit for preventing COVID-19	260	57.40
Living in a crowded, dense community	285	62.91
Perceived severity		
Elderly, children, and sick people affected most.	380	83.89
COVID-19 causes mental health issues.	308	67.99
Unaffordable medical expenses and inappropriate WASH facilities	301	66.45
Loss of job and income during COVID-19	329	72.63
Inadequate protection information and assistance	300	66.23

WASH = water, sanitation, and hygiene.

More than 60% of respondents perceived themselves to be vulnerable because they believed COVID-19 infection was associated with poor housing conditions, inadequate water, poor sanitation, and water pollution. A Myanmar migrant expressed concern: “*My house condition is [inappropriate] to fulfill COVID-19 guidelines and our community is a very small, densely populated area*.” A Cambodian woman noted “*I believe that access to safe water and sanitation can reduce COVID-19 transmission*.”

These dwellers also perceived the severity of the COVID-19 pandemic, which can lead to mental health problems, and loss of jobs and income. One Laotian man worried, “*COVID-19 is killing a million people. But most of us are daily wage workers. Yes . . . we are afraid of COVID-19, less than no food and loss of job*.” A Cambodian woman said, “*COVID-19 has made many people stressful and hopeless as we have to stay at home and do nothing. It affects our mental health*.” They considered the situation to be severe because they are not able to afford costly medical expenses and there is no access to appropriate WASH facilities. They also do not receive adequate COVID-19 information or social assistance. A Thai adolescent urged, “*We do not have access to fast and adequate information about COVID-19, and thus do not know how to protect ourselves*.”

### Slum dwellers’ awareness of and practices against COVID-19 infection.

Table 3 reports the percentage of respondents who have awareness regarding COVID-19 and its protection practices. Although only a small number of respondents know the nature and origin of the COVID-19 virus, most of them (> 75%) are aware of COVID-19 symptoms, the incubation period, and prevention and protection practices such as handwashing, wearing masks, and social distancing. The data show the high level of respondents’ awareness of COVID-19 and protection practices. Respondents declared they do not receive immediate and appropriate information, nor do they receive social assistance from governmental and nongovernmental organizations. However, they have learned from their slum community to cope with COVID-19. As one Thai woman noted, “*My community taught me how to prevent COVID-19 and behave in public. Our community is really [cohesive]*.” Religious communities also play role in raising awareness of slum communities in combating COVID-19. A Myanmar-Rohingya woman shared, “*Our religious community helps us to overcome the situation . . . and I believe that praying to God helps me to combat COVID-19*.”

**Table 3:** Urban slum dwellers’ awareness of and practices against contracting COVID-19

Awareness and practices	No. of agreed respondents	Frequency, % (*N* = 453)
Awareness of COVID-19		
COVID-19 is caused by a unique group of viruses transmitted from animal to human.	168	37.09
Signs of COVID-19 infection include respiratory symptoms, fever, cough, shortness of breath, and difficulty breathing.	340	75.06
The incubation period of COVID-19 (2–14 days)	357	78.81
Awareness of protection practices		
Handwashing, wearing mask, and social distancing limit COVID-19 transmission.	404	89.18
Indoor and outdoor disinfection disactivate the virus spread.	358	79.03
Segregating, recycling, and disposing of medical waste reduce risk of contracting the virus.	309	68.21
Daily practices of COVID-19 prevention		
Wash hands with soap regularly.	384	84.77
Avoid contact with sick people.	334	73.73
Practice social distancing of 2 m.	314	69.32
Wear mask in public.	375	82.78
Separate tissues/mask garbage at home.	221	48.79
Clean and disinfect household objects.	258	56.95
Stay a home when with fever.	348	76.82

Nearly 90% of respondents declared they wash their hands with soap and disinfectant regularly, wear masks in public (82%), avoid contact with sick people (> 73%), and stay home if they have a fever (76%). They are aware that wearing masks not only protects themselves, but also their community. One disabled woman said, “*I wear masks and wash my hands after coming home. I think those practices help us protect our health and community*.”

### Factors influencing slum dwellers’ threat appraisal, awareness, and protective practices.

Table 4 shows the sociodemographic characteristics of respondents that affect their perceived vulnerability to, severity of, awareness of, and practices against contracting COVID-19.

**Table 4:** Multi-regression analysis of factors affecting urban slum dwellers’ perceived vulnerability to, severity of, awareness of, and practices against contracting COVID-19

Characteristic	Perceived vulnerability		Perceived severity		Awareness		Practice	
	β	*P* value	β	*P* value	β	*P* value	β	*P* value
Age	0.182	< 0.001	0.033	0.59	0.052	0.40	0.125	0.04
Gender	–0.102	0.02	0.055	0.24	0.061	0.21	–0.113	0.01
Educational level	–0.081	0.13	0.07	0.207	0.136	0.02	0.077	0.17
Nationality	–0.173	< 0.001	–0.078	0.11	0.039	0.44	–0.114	0.02
Marital status	–0.024	0.62	–0.082	0.11	–0.091	0.08	–0.092	0.07
Residential occupancy	–0.105	0.02	–0.146	< 0.001	–0.112	0.02	–0.16	0.74
Occupation	–0.037	0.44	–0.24	< 0.001	–0.075	0.14	–0.134	0.00
Model *P* value	< 0.001		< 0.001		0.058		< 0.001	
*R*^2^ value	0.13		0.08		0.03		0.06	

Age did not affect respondents’ awareness, practices, or perceived severity, but did affect perceived vulnerability. The elderly perceived themselves as more vulnerable than younger people (β = 0.182, *P* < 0.001). There was no difference between men and women with regard to perceived severity and awareness of the COVID-19 pandemic, but women tended to perceive a greater vulnerability (β = –0.102, *P* = 0.02) and to practice better protection (β = –0.113, *P* = 0.02) than men. Education level affected respondents’ awareness positively (β = 0.136, *P* = 0.02). Respondents who had higher levels of education generally had a greater awareness of the COVID-19 pandemic and protective practices. Foreign migrants tended to be more vulnerable (β = –0.173, *P* < 0.001), but performed better protective practices (β = –0.1714, *P* = 0.02) than Thai respondents. Respondents from different types of residences had different levels of awareness (β = –0.105, *P* = 0.02). The type of residential occupancy affected respondents’ perceptions of vulnerability (β = –0.105, *P* = 0.02) and severity (β = –0.146, *P *< 0.001). People who live in unoccupied spaces, under tollways, and near railroads perceived greater vulnerability and severity than those who live in houses. Type of occupation also affected respondents’ perception of severity (β = –0.24, *P* < 0.000) and protection practices (β = –0.134, *P* = 0.01).

## DISCUSSION

Our findings show that slum dwellers in Bangkok, Thailand, perceived their vulnerability to and severity of COVID 19. Sociodemographic factors such as age, gender, nationality, occupation, and housing conditions affected how people perceived vulnerability to, severity of, awareness of, and practices against contracting COVID-19. Many of these individuals are unable to practice COVID-19 prevention because of poor housing conditions, limited access to clean water and sanitation, and unaffordable protective equipment.

WASH played a vital role in combating COVID-19,^7^ but slum communities all over the world still struggled with access to clean water, sanitation, personal protective equipment, and public health services during the pandemic. Many dwellers in the study area use water from the canals for washing because they are not able to afford tap water during the pandemic. The quality of water in vending machines and canals has been found to be unsafe, with pathogens and high Biochemical oxygen demand (BOD) values (≈52.6%), according to a report of the Office of Water Quality Management, Thai Ministry of the Interior.^8^

The difficulties encountered by slum communities in Bangkok during the pandemic are similar to those in many other developing countries.^9^^,^^10^ The slum dwellers in our study are mainly low-skilled migrant workers who lack adequate access to WASH and health-care services, and cannot afford protective medical equipment. They highly perceived their vulnerability to and severity of contracting COVID-19 because they live in densely populated areas with poor-quality housing, lack of adequate living space and public services, low daily earnings, and unstable jobs and lives. The elderly, women, foreign migrants, and people living in unoccupied spaces, under tollways, and near railroads were perceived as being more vulnerable to contracting COVID-19. Similar findings were found in other studies in which the elderly were perceived as more vulnerable than younger people because of their physical and mental health conditions.^11^ Elderly people had a self-perception of being at greater risk than young people because of psychosocial factors such as fear of death and anxiety.^12^ One study^13^ found that women perceived themselves to be more vulnerable to COVID-19 than men because women are often employed retail markets that are prone to disruption and environmental impacts^14^ and because they are family caregivers. Foreign migrants in our study perceived themselves as more vulnerable than local dwellers, which was also found by Niño et al.^15^ Foreign migrants perceived threats from the pandemic as a result of barriers to access health care and ethnic discrimination. Our study found that type of residential occupancy (living in unoccupied spaces, under tollways, and near railroads) was a significant factor for dwellers’ perceived vulnerability to COVID-19. Poor-quality housing and poor ventilation lead to mold, mites, and other allergens causing poor health and lack of basic WASH facilities, is a favorable environment for infectious disease spread.^16^ In addition, people with residential instability and unstable occupations (daily wage workers)^17^ had a high perception of the severity of COVID-19, because they might lose their living space, job, and income, resulting in hunger and homeless.

Because slum dwellers in this study had a high perception of their vulnerability to and severity of COVID-19, most have a good awareness COVID-19 symptoms and practice good protective techniques. Higher education levels and female gender were factors that influenced COVID-19 awareness in all ethnic groups, and women tended to follow COVID-19 protection guidelines better than men. Foreign ethnic groups and daily wage workers also practiced more COVID-19 protection than other groups. Awareness along with motivation and ability to understand and appraise health risks or vulnerable situations help people make better decisions regarding improving their health practices, preventing disease, and protecting their health.^18^ Perceived vulnerability can help people enhance preventive practices because of emotions such as fear and anxiety.^19^ Individual good preventive practices can help reduce the severity of harmful events and increase self-efficacy regarding disease prevention benefits.

## CONCLUSION

Our study depicted a realist picture of the lives of urban marginalized people who live in slums during the pandemic. People in these informal settlements are being deprived of their right to water and sanitation, with severe implications on their ability to prevent and survive COVID-19 infection. Despite the great efforts of international development organizations and governments in urban WASH and health-care programs, many slum communities exist in poor urban environments across the world. The results of our study note the need for urgent intervention and special assistance from development organizations, the government, and society in responding to the requirements of slum dwellers by ensuring access to clean water, sanitation, and health-care services. Any WASH and health programs responding to future pandemics must reflect slum dwellers’ sociodemographic characteristics to enhance their threat appraisal capacity and coping abilities. This requires the continuous engagement of international development organizations and governments with slum communities to identify possible barriers to disease prevention, access to public health-care services, and social protection. Efforts should also be made to improve the availability of immediate information about the ways to combat the pandemic, including both offline and online information platforms in different languages for different ethnic groups in slum communities.

## References

[1] UN Habitat , 2003. The Challenge of Slums: Global Report on Human Settlements. 2003. United Nations Human Settlements Programme. Earthscan Publications Ltd. London and Sterling, VA.

[2] Marx B Stoker T Suri T , 2013. The economics of slums in the developing world. J Econ Perspect 27: 187–210.

[3] World Bank , 2015. *Water Supply, Sanitation, and Hygiene (WASH) Poverty Diagnostic Initiative*. Available at: https://www.worldbank.org/en/topic/water/publication/wash-poverty-diagnostic. Accessed 9 July 2021.

[4] Wasdani KP Prasad A , 2020. The impossibility of social distancing among the urban poor: the case of an Indian slum in the times of COVID-19. Local Environ 25: 414–418.

[5] Baltar F Brunet I , 2012. Social research 2.0: virtual snowball sampling method using Facebook. Internet Res 22: 57–74.

[6] Creswell JW , 2009. Research Design: Qualitative, Quantitative, and Mixed Methods Approaches, 3rd edition. Thousand Oaks, CA: Sage Publications.

[7] UNICEF , 2020. *WASH Programme Contribution to Coronavirus Disease (COVID-19) Prevention and Response*. Available at: https://www.unicef.org/media/66091/file/UNICEF-WASH-COVID-19-prevention-response-overarching.pdf. Accessed 1 October 2021.

[8] Office of Water Quality Management , 2019. Pollution-Free Metropolitan Safety Audit Report: Department of Drainage and Sewerage. Bangkok, Thailand: Ministry of the Interior.

[9] Islam S Emran GI Rahman E Banik R Sikder T Smith L Hossain S , 2021. Knowledge, attitudes and practices associated with the COVID-19 among slum dwellers resided in Dhaka city: a Bangladeshi interview-based survey. J Public Health (Oxf) 43: 13–25. 33057666 10.1093/pubmed/fdaa182 PMC7665690

[10] Friesen J Pelz PF , 2020. COVID-19 and slums: a pandemic highlights gaps in knowledge about urban poverty. JMIR Public Health Surveill 6(3): e19578: 1–11. 32877347 10.2196/19578 PMC7486000

[11] Witte K Allen M , 2000. A meta-analysis of fear appeals: implications for effective public health campaigns. Health Educ Behav 27: 591–615. 11009129 10.1177/109019810002700506

[12] Pasion R Paiva TO Fernandes C Barbosa F , 2020. The age effect on protective behaviors during the COVID-19 outbreak: sociodemographic, perceptions and psychological accounts. Front Psychol 11: 561785: 1–14. 33178069 10.3389/fpsyg.2020.561785 PMC7595956

[13] Guo Y Qin W Wang Z Yang F , 2021. Factors influencing social distancing to prevent the community spread of COVID-19 among Chinese adults. Prev Med 143: 106385. 1–5. 33359017 10.1016/j.ypmed.2020.106385 PMC7774485

[14] Nguyen TPL Peña-García A , 2019. Users’ awareness, attitudes, and perceptions of health risks associated with excessive lighting in night markets: policy implications for sustainable development. Sustainability 11: 6091. 1–14.

[15] Niño M Harris C Drawve G Fitzpatrick KM , 2021. Race and ethnicity, gender, and age on perceived threats and fear of COVID-19: evidence from two national data sources. SSM Popul Health 13: 100717. 1–8. 33344747 10.1016/j.ssmph.2020.100717 PMC7733547

[16] Krieger J Higgins DL , 2002. Housing and health: time again for public health action. Am J Public Health 92: 758–768. 11988443 10.2105/ajph.92.5.758 PMC1447157

[17] Otto C Haller AC Klasen F Hölling H Bullinger M Ravens-Sieberer U , 2017. Risk and protective factors of health-related quality of life in children and adolescents: results of the longitudinal BELLA study. PLoS One 12: e0190363. 1–12. 29284054 10.1371/journal.pone.0190363 PMC5746247

[18] He Z Cheng Z Shao T Liu C Shao P Bishwajit G Feng D Feng Z , 2016. Factors influencing health knowledge and behaviors among the elderly in rural China. Int J Environ Res Public Health 13(10): 975. 1–16. 10.3390/ijerph13100975 PMC5086714 27706061

[19] Stangier U Kananian S Schüller J, 2021. Perceived vulnerability to disease, knowledge about COVID-19, and changes in preventive behavior during lockdown in a German convenience sample. Curr Psychol 2021: 1–9. 10.1007/s12144-021-01456-6 PMC7906828 33654348

